# Limited antimicrobial efficacy of oral care antiseptics in microcosm biofilms and phenotypic adaptation of bacteria upon repeated exposure

**DOI:** 10.1007/s00784-020-03613-w

**Published:** 2020-10-08

**Authors:** Sophia R. Schwarz, Stefanie Hirsch, Andreas Hiergeist, Christian Kirschneck, Denise Muehler, Karl-Anton Hiller, Tim Maisch, Ali Al-Ahmad, André Gessner, Wolfgang Buchalla, Fabian Cieplik

**Affiliations:** 1 Department of Conservative Dentistry and Periodontology, University Hospital Regensburg, Franz-Josef-Strauß-Allee 11, 93053 Regensburg, Germany; 2 Institute of Clinical Microbiology and Hygiene, University Hospital Regensburg, Regensburg, Germany; 3 Department of Orthodontics, University Hospital Regensburg, Regensburg, Germany; 4 Department of Dermatology, University Hospital Regensburg, Regensburg, Germany; 5 Department of Operative Dentistry and Periodontology, Center for Dental Medicine, Faculty of Medicine, University of Freiburg, Freiburg im Breisgau, Germany

**Keywords:** Antimicrobial, Biofilm, Microcosm, Antiseptic, Adaptation

## Abstract

**Objectives:**

The aims of this study were to investigate the antimicrobial efficacy of antiseptics in saliva-derived microcosm biofilms, and to examine phenotypic adaption of bacteria upon repeated exposure to sub-inhibitory antiseptic concentrations.

**Methods:**

Saliva-derived biofilms were formed mimicking caries- or gingivitis-associated conditions, respectively. Microbial compositions were analyzed by semiconductor-based 16S rRNA sequencing. Biofilms were treated with CHX, CPC, BAC, ALX, and DQC for 1 or 10 min, and colony forming units (CFU) were evaluated. Phenotypic adaptation of six selected bacterial reference strains toward CHX, CPC, and BAC was assessed by measuring minimum inhibitory concentrations (MICs) over 10 passages of sub-inhibitory exposure. Protein expression profiles were investigated by SDS-PAGE.

**Results:**

Both biofilms showed outgrowth of streptococci and *Veillonella* spp., while gingivitis biofilms also showed increased relative abundances of *Actinomyces*, *Granulicatella*, and *Gemella* spp. Antiseptic treatment for 1 min led to no relevant CFU-reductions despite for CPC. When treated for 10 min, CPC was most effective followed by BAC, ALX, CHX, and DQC. Stable adaptations with up to fourfold MIC increases were found in *E. coli* toward all tested antiseptics, in *E. faecalis* toward CHX and BAC, and in *S. aureus* toward CPC. Adapted *E. coli* strains showed different protein expression as compared with the wildtype strain.

**Conclusion:**

Antiseptics showed limited antimicrobial efficacy toward mature biofilms when applied for clinically relevant treatment periods. Bacteria showed phenotypic adaptation upon repeated sub-inhibitory exposure.

**Clinical relevance:**

Clinicians should be aware that wide-spread use of antiseptics may pose the risk of inducing resistances in oral bacteria.

## Introduction

The predominant mode of bacterial life is in biofilms, which mean highly structured, surface-associated communities of microorganisms that are embedded into self-produced extracellular substances (EPS) and exhibit an altered phenotype as compared with their planktonic counterparts [1, 2]. Accordingly, it is well known that the concentrations of antiseptics and antibiotics needed to kill sessile bacteria in biofilms are about 100 to 1000 times higher than the concentrations necessary to eradicate planktonic (i.e., “free-floating”) bacterial cells [3, 4].

Dental caries and periodontal diseases, which both are among the most prevalent non-communicable diseases worldwide [5], are associated with biofilms [6]. In the oral cavity, formation of biofilms (“dental plaque”) occurs on tooth or dental material surfaces, which is on the one hand essential for the physiology of the oral cavity, but can also result in clinical signs of dental caries or gingivitis, driven by emergence of dysbiosis in the oral microbiota [7]. While mechanical removal or disruption of oral biofilms and concomitant use of fluorides remain the cornerstones of contemporary oral hygiene concepts [8–10], this may not be sufficient for high-risk groups such as patients with mental disabilities [11], patients with fixed orthodontic appliances [12] or after periodontal or implant surgical procedures [13], or elderly patients in general [14]. In these cases, the use of antiseptic mouthwashes can be recommended as adjunct to mechanical biofilm removal, e.g., for managing gingivitis [15, 16] or in caries-active subjects [17, 18].

Currently, a wide range of antiseptic mouthwashes is available as over-the-counter consumer products, comprising various antiseptics or essential oils [15, 16, 19]. While the bis-biguanide chlorhexidine digluconate (CHX) is considered the gold-standard antiseptic in oral care [20, 21], also quaternary ammonium compounds (QACs) such as cetylpyridinium chloride (CPC), benzalkonium chloride (BAC) and dequalinium chloride (DQC), or the bis-biguanide alexidine dihydrochloride (ALX) have been investigated [15, 16, 19, 22].

Although it is well established that these antiseptics provide clinical benefits in terms of reducing oral biofilm formation and managing gingivitis (mostly by decreasing the salivary bacterial load), their antimicrobial efficacy toward preformed, mature oral biofilms may be limited when applied for clinically realistic treatment periods [21–23]. For instance, treatment with 0.2% CHX for 1 min only affected the outer layers of biofilms formed in situ for 48 h, as it was shown in a classic study by confocal laser scanning microscopy combined with LIVE/DEAD staining [24]. Therefore, clinical use of an antiseptic mouthwash may result in concentration gradients in oral biofilms, with lower biofilm strata being exposed to sub-inhibitory concentrations of the antiseptics [21, 22]. Upon multiple exposures to such sub-inhibitory concentrations, oral bacteria may be able to phenotypically adapt toward these antiseptics by employing analogous mechanisms as known for resistance against antibiotics [21, 22, 25–27]. Since the oral cavity may be considered potential reservoir of resistance genes, antimicrobial resistance may easily be spread among oral biofilm bacteria via horizontal gene transfer [28–30]. Interestingly, the potential risks associated with the widespread use of antiseptics in oral care have only been highlighted very recently [21, 22, 31, 32].

The aims of the present in vitro study were twofold: first, to investigate the antimicrobial efficacy of five antiseptics used in oral care (CHX, CPC, BAC, ALX, and DQC) toward microcosm biofilms cultured from human saliva, and second, to examine whether selected bacterial reference strains could phenotypically adapt upon repeated exposure to sub-inhibitory concentrations of CHX, CPC, and BAC.

## Material and methods

### Test substances

Chlorhexidine digluconate (CHX; Sigma C9394), benzalkonium chloride (BAC; Sigma 12,060), alexidine dihydrochloride (ALX; Sigma A8986), dequalinium chloride (DQC; Sigma PHR1300; all: Sigma-Aldrich, St. Louis, MO, USA), and cetylpyridinium chloride (CPC; Merck 6,002,006; Merck, Darmstadt, Germany) were chosen as antiseptics to be tested in the present study. CHX, BAC, CPC, and DQC were all solved in dH_2_O and diluted to the respective treatment concentrations (0.1% and 0.2% for CHX, 0.05% and 0.1% for CPC, BAC, and DQC). For ALX, a 1% stock solution was prepared in DMSO, which was then further diluted in dH_2_O to the treatment concentrations of 0.05% and 0.1%.

CHX and ALX are bis-biguanides that acquire two hydrogen ions from two gluconic acid (CHX) or dihydrochloride molecules (ALX), respectively, both becoming double-positively charged. CPC and BAC are QACs carrying a single positive charge, while DQC is a double-positively charged QAC.

### Saliva collection

A healthy, 23-year-old female (author SH) with no history of dental caries, periodontitis, or other oral diseases and no intake of antibiotics within the past 3 months volunteered for collection of saliva. After detailed description of the study outline, written informed consent was obtained. The medical ethical approval for the protocol was obtained from the ethics committee of the University of Regensburg (ref. 17-782_1-101).

The sampling took place on a single appointment between 9 and 11 am with the volunteer not having consumed anything on the respective day except water. Unstimulated saliva was collected using the spitting method [33]. The volunteer was asked to let saliva gather on the bottom of her mouth and spit into a tube every 30 s for a total period of 10 min. Immediately afterwards, the collected saliva was vortexed (REAX top, Heidolph Instruments, Schwabach, Germany; 35 kHz) for 10 s, placed in an ultrasonic water-bath chamber (Sonorex Super RK 102 H, Bandelin, Berlin, Germany) for 2 min, and vortexed again for 10 s in order to separate aggregated bacteria. Afterwards, saliva was diluted 1:1 in 60% glycerol and split into 1-mL aliquots that were immediately frozen at − 20 °C for later use for inoculation of biofilms.

### Inoculation and culture of saliva-derived microcosm biofilms

Biofilms were cultured in the so-called *Amsterdam Active Attachment* (AAA) model, which is a high-throughput biofilm model based on active attachment of bacteria to different substrates and has been described earlier in detail [34]. For the present study, the AAA model consisted of a custom-made stainless-steel lid with 24 clamps containing glass discs (diameter 12 mm; Menzel, Braunschweig, Germany) that fitted on top of a 24-well polystyrene microtiter plate (Corning^®^ Costar^®^, Corning, NY, USA), allowing for 24 individual biofilms to form.

As a basal nutrient broth, the complete saliva (CS) broth as described by Pratten et al. [35] was used and modified in two different ways: (1) by adding sucrose (final concentration: 0.1%) for mimicking caries-associated conditions (caries broth), and (2) by adding vitamin K_1_ (0.00002%), vitamin K_3_ (0.0001%), and hemin (0.1%) for mimicking gingivitis-associated conditions (gingivitis broth).

For the preparation of the inoculation medium, one stored saliva aliquot was thawed and 800 μL from this aliquot were mixed with 40 mL of caries broth or a 70%/30% mixture of gingivitis broth and heat-inactivated fetal bovine serum (FBS; Gibco® Qualified FBS, Thermo Fisher Scientific, Darmstadt, Germany), respectively, and vigorously vortexed. Then, 1.5 mL were added per each well of a 24-well plate and the models were subsequently incubated anaerobically (80% N_2_, 10% CO_2_, 10% H_2_) in a microincubator (MI23NK, SCHOLZEN Microbiology Systems, St. Margrethen, Switzerland) for 8 h for allowing initial attachment to the glass discs. After this initial attachment period, the lids containing the glass discs were carefully moved up and down to remove loosely bound bacteria and transferred to 24-well plates containing fresh caries broth or gingivitis broth, respectively. Medium was refreshed again after 24 h and 48 h of culture.

### Extraction of nucleic acids from saliva and biofilm samples

Biofilms were cultured as described above (*n* = 4 for each nutrient broth) and saliva aliquots (*n* = 4) were thawed. After the total biofilm culture period of 72 h, the glass discs were carefully removed from the lids using sterile forceps and transferred to sterile Eppendorf tubes containing 1 mL of 0.9% NaCl. Biofilm dispersal was ensured by vortexing for 10 s, placing in an ultrasonic water-bath chamber (35 kHz) for 10 min, and vortexing again for 10 s. Complete biofilm removal from the glass discs was confirmed visually.

Microbial nucleic acids were immediately stabilized by mixing biofilm and saliva samples 1:2 with magic PBI microbiome preservation buffer (microBIOMix GmbH, Regensburg, Germany). Stabilized samples were stored at − 80 °C until further processing. Mechanical cell disruption was used for pre-lysis of microbial cells by applying repeated bead beating. Therefore, at total of 500 μL stabilized sample material was added into lysing matrix B tubes (MP Biomedicals, Eschwege, Germany) and further processed in the TissueLyser II instrument (Qiagen, Hilden, Germany) at 60 Hz for 3 × 1 min. Nucleic acids were purified from total crude cell extracts by means of the MagNA Pure 96 instrument (Roche Diagnostics, Mannheim, Germany). Quantification of total nucleic acids was carried out by using the NanoDrop 1000 spectrophotometer (Thermo Fisher Scientific).

### Semiconductor-based sequencing of bacterial 16S rRNA genes

Copy numbers of bacterial 16S rRNA genes were quantified in nucleic acid extracts by a quantitative real-time PCR protocol, as described before [36], and were normalized to 1e+6 copies per mL. Subsequently, V1 to V3 hypervariable regions of bacterial 16S rRNA genes were amplified from a total of 1e+7 bacterial 16S rDNA copies with primer S-D-Bact-0008-c-S-20 containing a 10-bp barcode sequence and IonTorrent-specific sequencing adaptor A, and S-D-Bact-0517-a-A-18 containing a 3’-P1 adapter sequence using the Platinum II Taq Hot-Start DNA Polymerase (Thermo Fisher Scientific). After 30 PCR cycles, amplicons were purified twice with a 0.8 bead to DNA ratio using MagSi-NGS^PREP^ Plus beads (Steinbrenner Laborsysteme, Wiesenbach, Germany). Copy numbers of amplicons containing sequencing-adaptors were determined using the KAPA Library Quantification IonTorrent Kit (Roche Diagnostics) and pooled to equimolar amplicon concentrations of each sample. A total of 120 attomol of the final library pool was subjected to isothermal amplification with the Ion PGM™ Template IA 500 Kit before running 1100 flow cycles during high-throughput sequencing on an Ion Torrent™ S5 Plus machine (Thermo Fisher Scientific).

### Sequence processing and identification of amplicon sequence variants (ASVs)

Amplification primer and adapter sequences as well as low-quality bases were removed using cutadapt 1.24 and Trimmomatic 0.39. Cutadapt was also used for demultiplexing of filtered reads allowing no errors. All subsequent analyses were conducted with R 3.6.0. Here, resulting reads (36,144 ± 3172) were subjected to denoising sequencing data and generation of ASVs using dada2 (version 1.14.0). An unrooted phylogenetic tree was calculated with phangorn 2.5.5 after sequence alignment with DECIPHER 2.14.0 for later calculation of UniFrac distances with the phyloseq package. The IDTAXA algorithm and the All-Species Living Tree Project (LTP) reference database release 132 were used for taxonomic classification of ASVs. All plots were generated using the ggpubr 0.2.4 package.

### Antimicrobial assay

After the total biofilm culture period of 72 h, the lids containing the glass discs were moved to 24-well plates containing antiseptics (0.1% CHX, 0.2% CHX, 0.05% CPC, 0.1% CPC, 0.05% BAC, 0.1% BAC, 0.05% ALX, 0.1% ALX, 0.05% DQC, 0.1% DQC; 2 wells each) or 0.9% NaCl (4 wells) for treatment periods of 1 or 10 min, respectively. After the respective treatment period, the lid was removed and transferred to a new plate containing 0.9% NaCl for 5 s. Afterwards, the glass discs were carefully removed from the lids using sterile forceps and transferred to sterile Eppendorf tubes containing 1 mL of 0.9% NaCl. Biofilm dispersal was ensured by vortexing for 10 s, placing in an ultrasonic water-bath chamber (35 kHz) for 10 min and vortexing again for 10 s. Complete biofilm removal from the glass discs was confirmed visually. Then, tenfold serial dilutions (10^−1^ to 10^−7^) were prepared in 0.9% NaCl and aliquots (180 μL) were plated on Schaedler blood agar plates and incubated anaerobically for 72 h. Afterwards, colony forming units (CFUs) were evaluated. CFU data was analyzed using SPSS, version 25 (SPSS Inc., Chicago, IL, USA) and medians, 1st and 3rd quartiles, were calculated from at least five independent duplicate experiments for each treatment modality. The CFU reduction rates were calculated as follows:

$$ CFU\  reduction\ rate={\mathit{\log}}_{10}\ \left(\frac{median\  CFU\  of\ untreated\ control\ group}{median\  CFU\  of\ respective\ test\ group}\right) $$

Median CFU reduction rates by ≥ 3 log_10_ (99.9%) or by ≥ 5 log_10_ (99.999%) were regarded as biologically relevant antimicrobial activity or disinfecting effect, respectively [37, 38].

### Minimal inhibitory concentration (MIC) passaging and re-evaluation of phenotypic adaptation

Six reference strains, *Actinomyces naeslundii* (DSM 43013), *Enterococcus faecalis* (ATCC 29212), *Escherichia coli* (ATCC 25922), *Fusobacterium nucleatum* (DSM 20482), *Staphylococcus aureus* (ATCC 29213), and *Streptococcus mutans* (DSM 20523) were obtained from DSMZ (Deutsche Sammlung von Mikroorganismen und Zellkulturen, Braunschweig, Germany) and ATCC (American Type Culture Collection, Manassas, VA, USA). *A. naeslundii and F. nucleatum* were grown in modified fluid universal medium (mFUM) and on Schaedler agar, *E. faecalis* and *S. mutans* in brain heart infusion (BHI) broth (Sigma-Aldrich) and on BHI agar, and *E. coli* and *S. aureus* in Müller-Hinton (MH) broth and on MH agar (all agar plates were provided by the Institute for Clinical Microbiology and Hygiene, University Hospital Regensburg, Germany). For preparation of planktonic cultures, colonies were picked, suspended in 5 mL of the respective culture broth and cultured overnight at 37 °C for yielding bacteria in the stationary growth phase. *A. naeslundii and F. nucleatum* were cultured under anaerobic conditions (80% N_2_, 10% CO_2_, and 10% H_2_) in a microincubator (MI23NK, SCHOLZEN Microbiology Systems, St. Margrethen, Switzerland), while *E. faecalis*, *S. mutans*, *E. coli*, and *S. aureus* under aerobic conditions. Wild-type (WT) cultures were stored at − 80 °C in cryo banks (Mast Diagnostica Labortechnik, Reinfeld, Germany) for further analyses.

CHX, BAC, and CPC were chosen as antiseptics for these experiments. Twofold serial dilutions were prepared from stock solutions in the respective nutrient broth yielding CHX concentrations from 62.5 to 0.49 μg/mL, BAC concentrations from 90 to 0.71 μg/mL, and CPC concentrations from 84.8 to 0.66 μg/mL. MICs were examined for CHX, BAC, and CPC over 10 passages by employing a modified broth microdilution method: An overnight culture of the respective strain was adjusted to an optical density (OD) of 0.1, as measured with a spectrophotometer at 600 nm (Ultrospec 3300; Amersham Biosciences, Amersham, UK). Two hundred fifty microliters of these bacterial suspensions were added to wells of a 48-well polystyrene microtiter plate (Corning^®^ Costar^®^) that contained 250 μL of the respective antiseptic in the varying concentrations yielding an end volume of 500 μL in each well. After incubation at 37 °C for 24 h, the MICs were determined by visual examination. The well with the highest antiseptic concentration that still exhibited bacterial growth (turbidity) was defined as sub-MIC. The content of this sub-MIC well was added to 5 mL of fresh nutrient broth without antiseptic and incubated at 37 °C overnight. Then, a second passage of MIC evaluation and re-growth was performed as described before. This procedure was performed for 10 passages (P1 to P10) with six independent replicates each. Replicates that showed higher MICs at P10 as compared with P1 were stored at − 80 °C for further experiments. For evaluating stability of phenotypic adaptation, the frozen P10 cultures were thawed and cultured in fresh nutrient broth without antiseptic over-night at 37 °C. Afterwards, MICs were examined as described above (re-evaluation, R). This procedure was repeated twice in triplicates.

### Protein expression profiles of adapted P10 and WT *E. coli* strains

Protein expression profiles of adapted P10 and WT cultures were exemplarily investigated for *E. coli* by means of SDS-PAGE. WT and P10 cultures (*n* = 3) were grown in 5-mL MH broth overnight at 37 °C. Cells were harvested by centrifugation at 10,000×*g* for 5 min. After washing once with phosphate-buffered saline (PBS; Sigma-Aldrich), the pellet was resuspended in 100 μL NZY Bacterial Cell Lysis Buffer (Nzytech, Lisbon, Portugal) and incubated at 4 °C overnight. After overnight lysis, samples were centrifuged at 10,000×*g* for 5 min, and the supernatants were used for bicinchoninic acid assay (BCA assay) for determining protein concentrations. For SDS-Page, 8% separation gels and 5% stacking gels were prepared. Protein samples (20 μg) were mixed with Laemmli buffer (6x; Thermo Fisher Scientific), heated at 95 °C for 5 min, and cooled on ice for 10 min. After centrifugation, samples and a molecular weight marker (PageRuler™ Prestained Protein Ladder, 10 to 180 kDa; Thermo Fisher Scientific) were loaded on the polyacrylamide gels on separate lanes. Electrophoresis (Bio-Rad, Hercules, CA, USA) was performed at 70 V for 15 min at beginning and thereafter at 110 V for 115 min. Following electrophoresis, the gels were stained in Coomassie Blue R-250 staining solution (0.05%) for 2 h in the dark. All excess stain was washed out for 1 h, and gels were stored in acetic acid (1%) overnight. Afterwards, protein expression profiles were examined visually and representative protein expression profiles were photo-documented.

## Results

### Microbial compositions of saliva inoculum and caries or gingivitis biofilms

A total of 696 (mean 152 ± 146 per sample) amplicon sequence variants (ASVs) were detected by high-throughput sequencing of V1 to V3 variable regions of bacterial 16S rRNA genes.

Species richness represented by the number of detected ASVs was significantly lower in caries (mean 74 ± 16) and gingivitis (mean 52 ± 10) biofilms as compared with the saliva inoculum (mean 394 ± 43), with no significant differences between both biofilms.

**Fig. 1:**
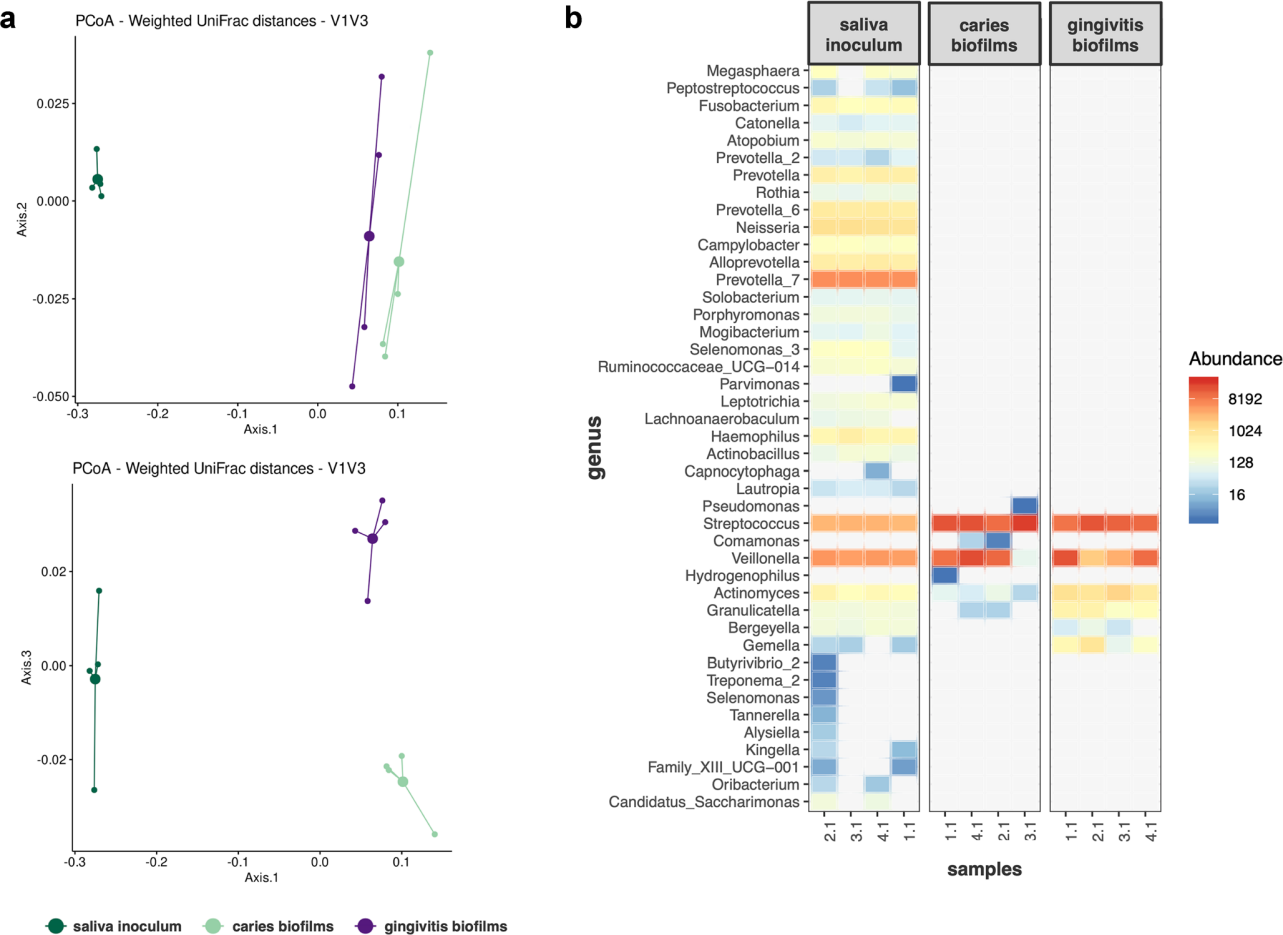
**a** Compositional differences of saliva inoculum, caries biofilms and gingivitis biofilms evaluated by Principle Coordinates analysis (PCoA) of weighted UniFrac distances. Depicted are principal coordinates 1 to 3, which explained 93% of total variance. Each sample is represented by a single dot; cluster centroids are highlighted by bold points. **b** Heatmap of ASV abundance on genus-level for saliva inoculum, caries biofilms and gingivitis biofilms

Also, microbial compositions based on weighted UniFrac distances (Fig. 1a) showed significant differences between saliva inoculum and caries biofilms (Adonis R2 = 0.81, padj = 0.03) and between saliva inoculum and gingivitis biofilms (Adonis R2 = 0.88, padj = 0.03). Likewise, there was a significant difference in microbial composition between caries and gingivitis biofilms (Adonis R2 = 0.7, padj = 0.03).

Figure 1b depicts a heatmap of ASV-abundance on genus level for saliva inoculum and both biofilms. The saliva inoculum shows a diverse microbial composition with *Prevotella*, *Streptococcus*, *Veillonella*, and *Neisseria* spp. being most abundant. The caries biofilms mainly comprised *Streptococcus* and *Veillonella* spp., which strongly increased in relative abundance as compared with the saliva inoculum. Gingivitis biofilms also showed an increase in abundance of *Streptococcus* and *Veillonella* spp., accompanied by increased relative abundance of *Actinomyces*, *Granulicatella*, and *Gemella* spp.

### Antimicrobial assay

**Fig. 2:**
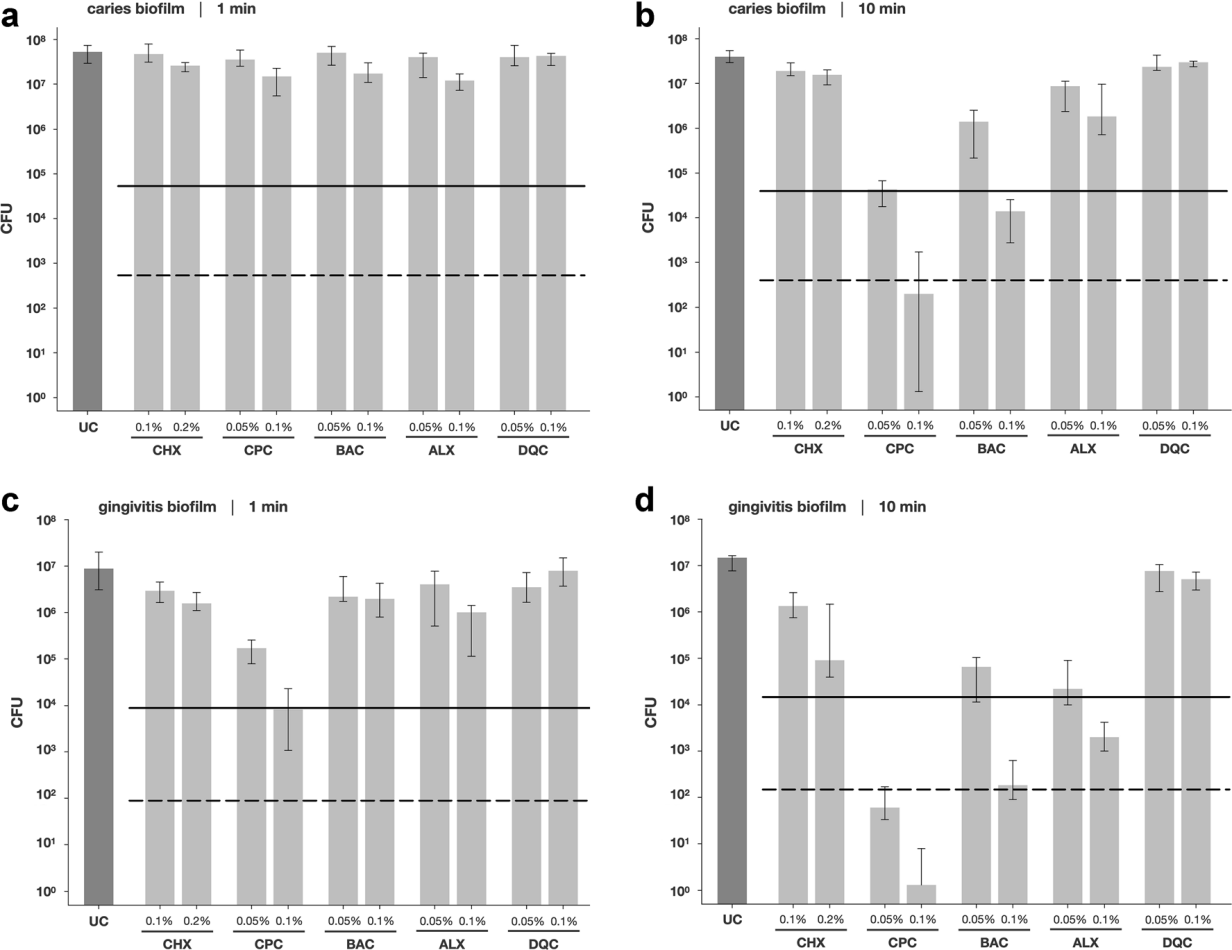
Antimicrobial efficacy of the tested antiseptics CHX, CPC, BAC, ALX, and DQC toward microcosm biofilms cultured in caries broth after treatment for 1 min (**a**) or 10 min (**b**) or microcosm biofilms cultured in gingivitis broth after treatment for 1 min (**c**) or 10 min (**d**), respectively. All results are depicted as medians, 1st and 3rd quartiles from at least five independent experiments, each performed in duplicate, on a log_10_-scaled ordinate. Horizontal dotted and dashed lines depict CFU reductions of 3 log_10_ and 5 log_10_, respectively, as compared with the untreated control UC

Figure 2 shows the results of the antimicrobial assay with CHX, CPC, BAC, ALX, and DQC toward microcosm biofilms cultured in caries or gingivitis broth for 72 h. In caries biofilms, all tested antiseptic exhibited CFU-reductions by < 1 log_10_ step when applied for a treatment period of 1 min (Fig. 2a). Upon treatment for 10 min, CPC exhibited CFU-reductions by 2.9 or 5.5 log_10_ at 0.05% or 0.1%, respectively. BAC reduced CFU by 1.7 or 3.7 log_10_ at 0.05% or 0.1%, respectively, and ALX 0.1% by 1.6 log_10_, while all other antiseptics exhibited CFU-reductions by < 1 log_10_ step (Fig. 2b).

In gingivitis biofilms, CPC reduced CFU by 1.9 (0.05%) or 3.1 log_10_ (0.1%) upon treatment for 1 min, while all others showed CFU-reductions by < 1 log_10_ step (Fig. 2c). Upon treatment for 10 min, CHX showed CFU-reductions by 1.1 (0.1%) or 2.4 log_10_ (0.2%), CPC by 5.7 (0.05%) or 7.1 log_10_ (0.1%), BAC by 2.6 (0.05%) or 4.9 log_10_ (0.1%), and ALX by 2.9 (0.05%) or 4.0 log_10_ (0.1%), while DQC exhibited CFU-reductions by < 1 log_10_ step (Fig. 2d).

### MIC passaging and re-evaluation of phenotypic adaptation

**Fig. 3:**
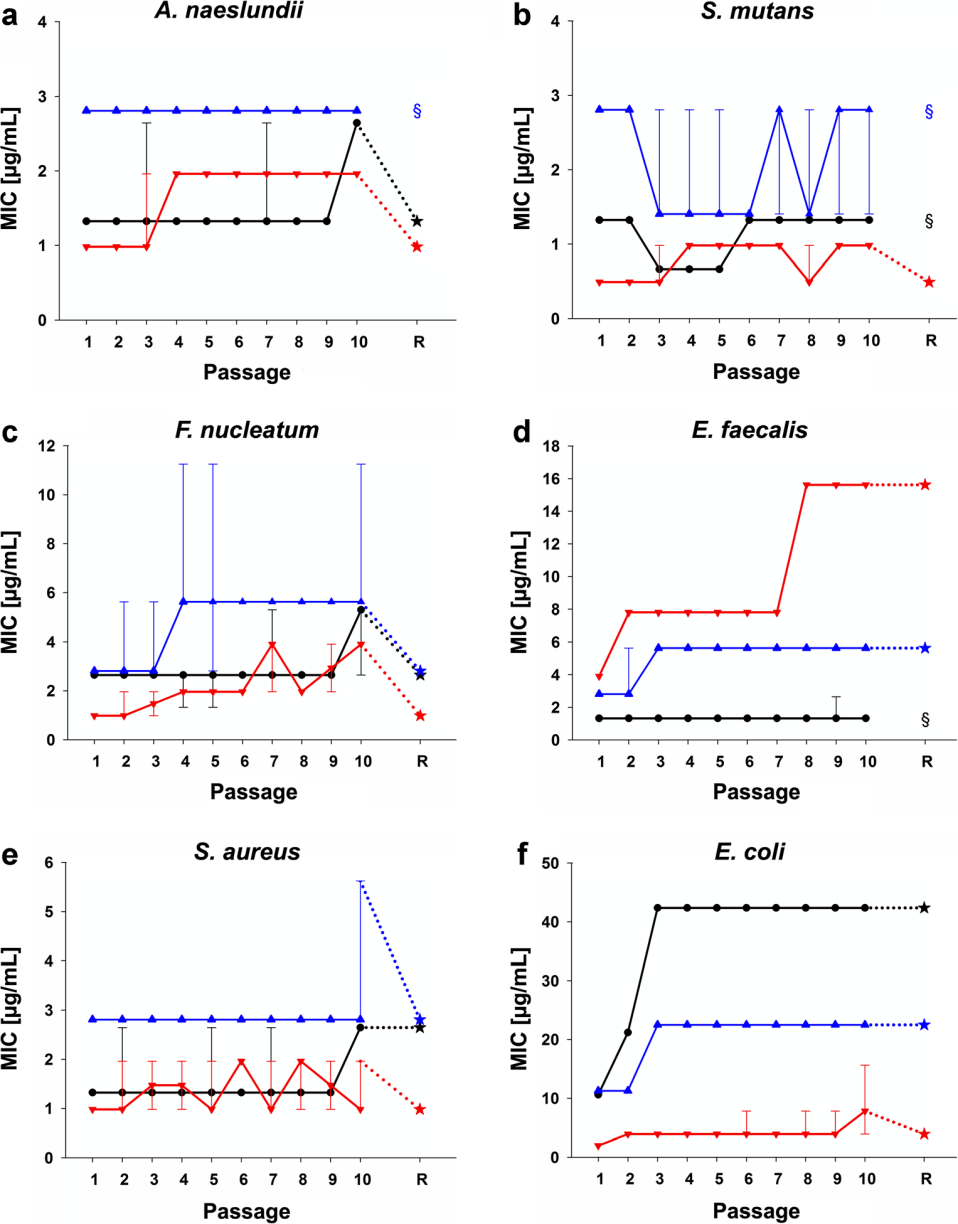
Phenotypic adaptation of *A. naeslundii* (**a**), *S. mutans* (**b**), *F. nucleatum* (**c**), *E. faecalis* (**d**), *S. mutans* (**e**), and *E. coli* (**f**) toward CHX (red), CPC (black), and BAC (blue). The ordinates show the respective MICs in μg/mL; the abscissae reflect the passages P1 to P10 and the re-evaluation R. All MICs are depicted as medians, min and max from the values of six independent (P1 to P10) or three independent duplicate (R) experiments. Asterisks depict MICs at R, while paragraphs indicate that no R was performed due to no MIC increase between P1 and P10

The results of the MIC passaging are shown in Fig. 3. *A. naeslundii* (Fig. 3a), *S. mutans* (Fig. 3b), and *F. nucleatum* (Fig. 3c) did not show increased MICs for any of the three antiseptics at P10 that stayed stable at re-evaluation (R). *E. faecalis* (Fig. 3d) showed a twofold MIC increase from 2.8 (P1) to 5.6 μg/mL (P10) for BAC and a fourfold MIC increase from 3.9 (P1) to 15.6 μg/mL (P10) for CHX that both stayed stable at R. *S. aureus* (Fig. 3e) exhibited a twofold MIC increase from 1.3 (P1) to 2.6 μg/mL (P10) for CPC, which stayed stable at R. *E. coli* (Fig. 3f) exhibited a twofold MIC increase for BAC (P1: 11.3 μg/mL; P10: 22.5 μg/mL) and fourfold MIC increases for CHX (P1: 2.0 μg/mL; P10: 7.8 μg/mL) and CPC (P1: 10.6 μg/mL; P10: 42.4 μg/mL). P10-MICs remained stable at R for BAC and CPC and decreased for CHX to 3.9 μg/mL.

### Protein expression profiles of adapted P10 and WT *E. coli* strains

**Fig. 4:**
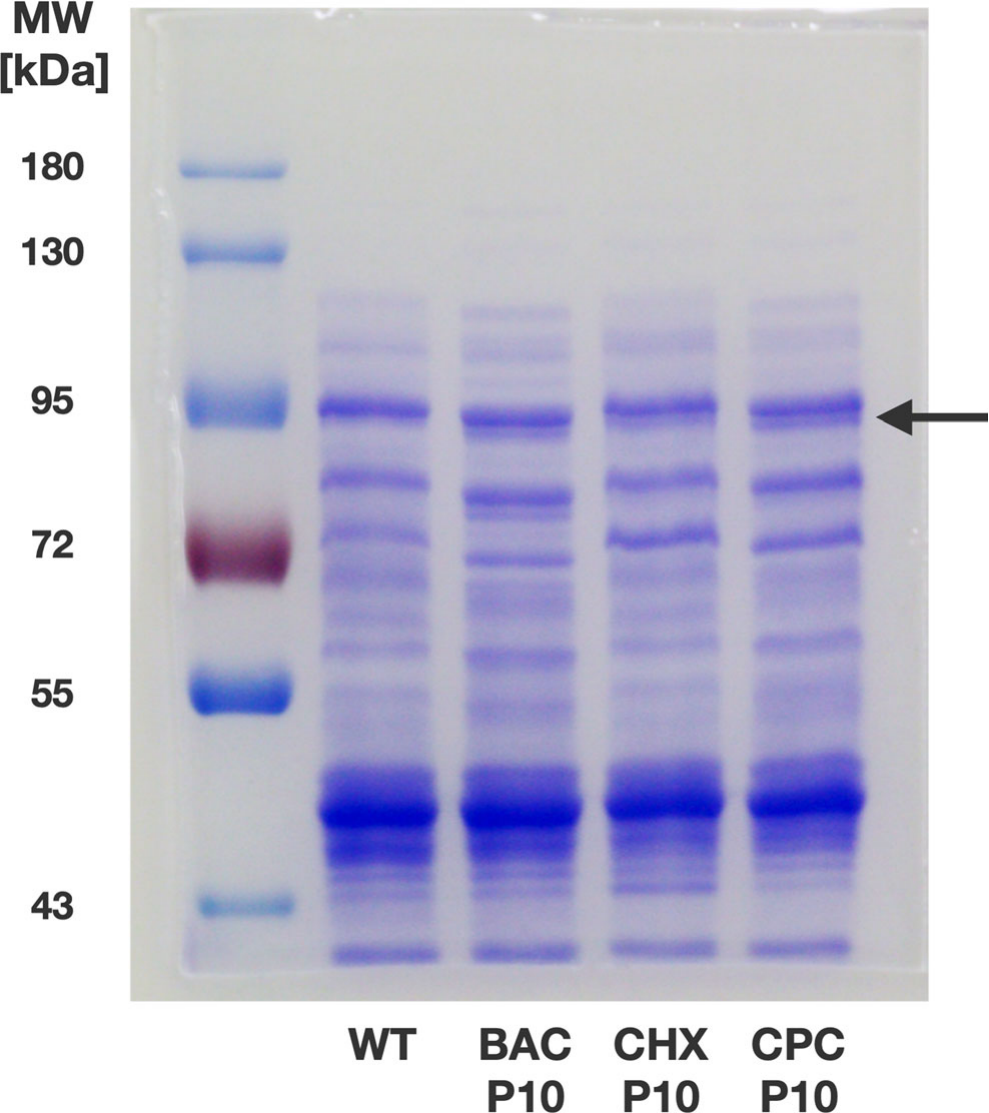
Protein expression profile of WT and adapted *E. coli* strains as shown by SDS-PAGE. A molecular weight (MW) marker is shown on the left. The black arrows points on an additional protein band slightly below the 95-kDa band that was found in all three adapted strains. The protein bands between the 95-kDa band and 55-kDa band of the BAC P10 strain exhibit a tendency for a “down-shift” as compared with the protein expression profiles of the other three strains

Since *E. coli* showed stable phenotypic adaptation toward all three antiseptics at R, protein expression profiles were investigated by SDS-PAGE for the adapted P10 strains (BAC P10, CHX P10, and CPC P10) as compared with the WT strain. A representative 8% SDS-PAGE gel is shown in Fig. 4. All three phenotypically adapted P10 strains presented an additional protein band slightly below the 95-kDa band, which could not be detected in the WT strain. Furthermore, there was a tendency for a “down-shift” of the protein bands between the 95-kDa band and 55-kDa band of the BAC P10 strain as compared with the protein expression profiles of the other three strains.

## Discussion

Antiseptic mouthwashes are available as over-the-counter products for consumers in order to be used adjunctively to mechanical biofilm removal as part of their daily oral care regimens [15–18]. The present in vitro study sought to investigate the antimicrobial efficacy of the antiseptics CHX, CPC, BAC, ALX, and DQC toward preformed, mature oral biofilms as they may be found in areas that are not entirely accessible to mechanical measures, such as interproximal or subgingival tooth surfaces [39].

For this purpose, microcosm biofilms were inoculated from human saliva and grown in vitro using the so-called *Amsterdam Active Attachment* (AAA) model. Microcosm biofilms are regarded to be closer to the complex situation found in vivo as compared with biofilm models from defined consortia (comprising a few different strains), while also exhibiting easier handling due to the in vitro-culture and less dependence on the compliance of study participants as compared with biofilms grown in situ on intra-oral appliances [34, 40, 41]. The AAA model offers the advantage of active attachment (rather than sedimentation) of the bacteria onto the substrate, and also facilitates controlling the periods which the biofilms are exposed to the tested compounds [34]. Choice of inoculum and growth conditions are crucial aspects for microcosm biofilms [40, 42–45]. In the present study, human saliva was chosen as inoculum source because it can be collected easier and in higher quantities as compared with dental plaque [40]. Furthermore, we could recently show that the choice of inoculum donors may be more important than the choice of the respective niche in those donors (e.g., saliva, subgingival plaque, or tongue scrapings) [40]. In contrast to this previous study, which aimed to mimic periodontitis-associated microbial communities [40], we chose to sample from one healthy donor here for growing biofilms that resemble microbial communities in a rather early stage of dysbiosis. Sampling from only one donor may be a drawback and potentially has an influence on the results because there may be donor-dependent effects with regard to the antimicrobial susceptibility of the biofilms, as it was recently shown by Chatzigiannidou et al. for microcosm biofilms from tongue swabs and treatment with CHX [45]. Therefore, it may be worthwhile to investigate the effects of different individual donors on the antimicrobial efficacy of antiseptics in the future. For mimicking caries- or gingivitis-associated conditions, a basal nutrient broth originally designed to mimic human saliva [35] was modified by adding sucrose or by adding vitamin K, hemin, and 30% serum, respectively [7]. Accordingly, the 16S rRNA sequencing results yielded a strong microbial shift with strongly reduced diversity and outgrowth of streptococci and *Veillonella* spp. as compared with the saliva inoculum when the biofilms were cultured in the caries broth. This effect can be related to the addition of sucrose, because streptococci ferment carbohydrates to lactic acid, while *Veillonella* spp., which play a crucial role in oral biofilm development [46], can utilize lactate and metabolize it to weaker acids such as propionate [47]. The microbial composition found in the gingivitis biofilms also exhibited outgrowth of streptococci and *Veillonella* spp., which was however accompanied with increased relative abundances of *Actinomyces* spp., *Granulicatella* spp., and *Gemella* spp. The latter three genera are among the most abundant taxa found in the oral cavity [48], but have also been linked to experimental gingivitis [49]. Still, the microbial composition found in the gingivitis biofilms does not represent gingivitis-associated microbial communities, which mainly comprise proteolytic taxa due to the change in environmental conditions involving high supply of proteins, mostly by increased secretion of gingival crevicular fluid [7]. This may be explained by the rather short total culture period of 72 h, which may not have provided enough time for fastidious bacteria to get established in the biofilms, while the daily supply of fresh nutrient broth may have fostered outgrowth of fast-proliferating taxa such as streptococci [40, 42, 43].

The oral care antiseptics CHX, CPC, BAC, ALX, and DQC were evaluated in two clinically relevant concentrations each and applied to both caries and gingivitis biofilms for either 1 or 10 min, which was meant to resemble the clinical use of a mouthwash or of an oral care gel, respectively [50]. In these set of experiments, CPC was found to be the most effective antiseptic, followed by BAC, ALX, and CHX in descending order with respect to their antimicrobial properties, while DQC showed no effects at all. These distinct antimicrobial efficacy rates may be explained by the respective chemical structures of the tested antiseptics and potential interactions with the biofilm matrix, the so-called extracellular polymeric substances (EPS), which may act as diffusion barrier for the antiseptics [2]. Accordingly, it is well known that cationic molecules like the ones tested here undergo electrostatic interactions, reactions, and sorption with matrix components, particularly with negatively charged EPS residues, limiting and retarding their penetration throughout the biofilm structure [51]. Furthermore, the rate of penetration decreases as a function of size (i.e., molecular weight and corresponding steric hindrance) [52]. The most effective compounds CPC and BAC both have a molecular weight of 304.5 Da, whereas the molecular weights of the less effective CHX (505.4 Da), ALX (508.8 Da), and DQC (456.7 Da; all molecular weights excluding counter ions) are considerably higher. Furthermore, CPC and BAC are single-positively charged, while CHX, ALX, and DQC carry two positive charges. Therefore, CPC and BAC may be hindered in their penetration to a lesser degree as compared with the larger and double-positively charged molecules, although not only cationic moieties like the quaternary ammonium groups but also alkyl chains may interact with EPS residues in terms of hydrophobic interactions [53].

Antimicrobial efficacy rates are in general difficult to compare between different in vitro biofilm models, mainly due to the vastly different biofilm culture protocols [54]. For instance, Voos et al. reported CFU-reduction by < 1 log_10_ after treating 72-h microcosm biofilms with CHX 0.2% for 3 min [55], while we found in a previous study that CHX 0.2% and CPC 0.1% achieved CFU reduction rates of about 5 log_10_ steps when applied for 10 min to polymicrobial 72-h biofilms cultured in vitro from *Actinomyces naeslundii*, *Actinomyces odontolyticus*, and *Streptococcus mutans* [50]. Accordingly, microcosm biofilms like in the present study are considered to resemble the physicochemical, microbiological, and nutrient conditions that are found in situ better than other in vitro biofilm models [41], which may also be reflected in smaller antimicrobial efficacy rates found here, hinting to high general “robustness” of these biofilms. Interestingly, the caries biofilms were found to be more tolerant in general as compared with the gingivitis biofilms. The addition of sucrose to the caries nutrient broth may have fostered EPS-production in the caries biofilms [2, 56]. Particularly, streptococci, which were found to be the most abundant taxon in both biofilms, are well-known to be able to produce insoluble extracellular polysaccharides from sucrose, e.g., by glucosyl- and fructosyl-transferases in *S. mutans* [57]. Therefore, the enhanced tolerance of bacteria found in the caries biofilms may be mostly due to a distinct, potentially “stickier” and more dense EPS structure due to the differences in nutrient supply, which may have further impeded penetration of the positively charged antiseptics [2].

Given the results found in the antimicrobial assay, it seems reasonable that bacteria in deeper layers of oral biofilms will be exposed to sub-inhibitory concentrations after clinical application of an antiseptic mouthwash [21]. Particularly, at the shorter treatment period of 1 min, which resembles the clinical use of a mouthwash, no relevant CFU reductions (< 1 log_10_ step) were found in both biofilms for all antiseptics despite CPC. Therefore, the second part of this study sought to examine whether bacteria could phenotypically adapt toward antiseptics upon repeated exposure to such sub-inhibitory concentrations. Here, we focused on CHX, CPC, and BAC, and evaluated six bacterial reference strains. *A. naeslundii*, *F. nucleatum*, and *S. mutans* strains were chosen as typical oral bacteria, while *S. aureus*, *E. faecalis*, and *E. coli* were selected as typical quality control strains for antimicrobial susceptibility testing [58]. Despite clear break-point concentrations for determining bacterial resistance toward antibiotics, suchlike frameworks are not existent for antiseptics, which severely hampers interpretation of the relevance of phenotypic adaptations such as the ones found here [21, 22, 59]. According to Chapman et al., a parallel definition of resistance has evolved for antiseptics, which defines resistance as measurable MIC increase by a factor of four- to sixteen-fold upon repeated exposure [59]. In the present study, 10 passages comprising MIC measurements and re-growth of the sub-MIC populations in antiseptic-free nutrient broth (for stopping the selection pressure between the respective MIC evaluations) were carried out, and the strains exhibiting MIC increases between P1 and P10 were re-evaluated for the stability of this phenotypic adaptation. The tested *A. naeslundii*, *F. nucleatum*, and *S. mutans* strains showed no stable adaptations toward any of the tested antiseptics. On the contrary, classic studies from the 1970s reported that oral bacteria (particularly streptococci) were able to adapt toward antiseptics like CHX after long-term clinical use of CHX-containing mouthwashes or gels [60–62]. Therefore, different strains or clinical oral isolates of *Actinomyces* spp., *Fusobacterium* spp., or streptococci should be subjected to similar experiments in the future in order to obtain more reliable insights into the clinical relevance of a potential adaptation toward antiseptics in oral bacteria. On the contrary, we found stable adaptations with up to fourfold MIC increases in *E. coli* toward all three tested antiseptics, in *E. faecalis* toward CHX and BAC, and in *S. aureus* toward CPC. This is in line with Kitagawa et al. who found an about fourfold increased MIC for CHX in *E. faecalis* upon 10 passages of MIC testing and re-growth [31]. Likewise, Wang et al. showed a fourfold MIC-increase in *E. faecalis* and twofold MIC-increases in *F. nucleatum*, *Streptococcus gordonii*, and *Porphyromonas gingivalis* toward CHX upon 10 suchlike passages [63]. Employing a similar methodology, Verspecht et al. reported 1.3- to 5.5-fold MIC increases toward CHX and CPC in *Aggregatibacter actinomycetemcomitans*, *F. nucleatum*, *P. gingivalis*, *Prevotella intermedia*, *S. mutans*, and *Streptococcus sobrinus*, depending on the bacterial strain and the tested antiseptic [32]. However, these three studies evaluated phenotypic adaptation by exerting continuous selection pressure due to constant presence of the antiseptic [31, 32, 63]. In contrast, we cultured the strains overnight in antiseptic-free nutrient broth after each MIC-investigation in order to pause the selection pressure between the individual MIC investigations resembling a clinic-like situation. Fitness of adapted isolates is known to be a crucial point for emergence of resistance [64]: a resistant strain will only be able to outcompete its WT strain if it is able to replicate at least as fast as the respective WT strain [64]. While Wang et al. found that their adapted *S. gordonii* strain showed a decelerated growth rate as compared with the WT strain [63], the methodology of the present study ensured that the found phenotypic adaptations only included cases where the adapted strains showed no reduced fitness as compared with their respective WT strains.

The results of the present study suggest that phenotypically adapted strains exhibiting decreased susceptibility toward BAC, CHX, or CPC may emerge upon repeated exposure toward those antiseptics at sub-inhibitory concentrations. However, the underlying mechanisms in oral bacteria have only roughly been investigated so far [21, 22]. As *E. coli* was the only strain that showed stable adaptation toward all three tested antiseptics here, it was chosen for analysis of the protein expression profiles of the adapted strains as compared with the WT strain. All three adapted strains exhibited an additional protein band slightly below the 95-kDa band which clearly indicates adaptations in terms of altered protein expression as compared with the WT strain. Furthermore, the BAC P10 strain also showed a tendency for a “down-shift” of the protein bands between the 95-kDa band and 55-kDa band, which may be an indication for protein activation by regulated proteolysis [65]. Kitagawa et al. also examined protein expression profiles of their CHX-adapted *E. faecalis* strain and its WT strain by SDS-PAGE and found an additional 19-kDa band in the protein expression of the adapted strain [31], which had previously been found in vancomycin-resistant enterococci [66]. Verspecht et al. analyzed the proteomes of their adapted and WT strains and found that antiseptic-adapted bacteria changed their metabolic profiles by upregulation of proteins involved in energy metabolism and in amino acid, nucleotide, and inorganic ion metabolisms [32], which has analogously been described for some *S. aureus* and *Enterococcus* spp. that were resistant to various clinically relevant antibiotics [67]. Furthermore, increases in cell surface hydrophobicity were reported in antiseptic-adapted strains [31, 32]. Due to membrane-disrupting mechanism of action of CHX, CPC, and BAC, it seems reasonable that expression of efflux pumps or changes in the membrane composition may be potential resistance mechanisms against those agents [21]. This will have to be elucidated in further studies in greater detail.

## Conclusion

This study indicates that antiseptics may only have limited antimicrobial efficacy toward mature oral biofilms when applied for clinically relevant treatment periods. Therefore, bacteria in deeper biofilm layers may be exposed to sub-inhibitory concentrations. Bacteria are able to phenotypically adapt toward antiseptics upon repeated exposure to such sub-inhibitory concentrations. Future studies will have to investigate whether the widespread use of antiseptics in oral care may lead to emergence of antiseptic-resistant strains in oral biofilms and, potentially, also to the development of cross-resistances in these bacteria.
